# Design of Therapeutic Self-Assembled Monolayers of Thiolated Abiraterone

**DOI:** 10.3390/nano8121018

**Published:** 2018-12-07

**Authors:** Elżbieta U. Stolarczyk, Katarzyna Sidoryk, Marcin Cybulski, Marek Kubiszewski, Krzysztof Stolarczyk

**Affiliations:** 1 R&D Analytical Department, Pharmaceutical Research Institute, 8 Rydygiera Street, 01-793 Warsaw, Poland; e.stolarczyk@ifarm.eu (E.U.S.); m.kubiszewski@ifarm.eu (M.K.); 2 Chemistry Department, Pharmaceutical Research Institute, 8 Rydygiera Street, 01-793 Warsaw, Poland; k.sidoryk@ifarm.eu (K.S.); m.cybulski@ifarm.eu (M.C.); 3 Faculty of Chemistry, University of Warsaw, 1 Pasteura Street, 02-093 Warsaw, Poland

**Keywords:** self-assembled monolayer, thiolated abiraterone, thioglycolic acid (GA), electrochemistry, gold electrode, capacity, oxygen reduction

## Abstract

The aim of our work was to synthetize of a new analogue of abiraterone—thiolated abiraterone (HS-AB) and design a gold surface monolayer, bearing in mind recent advances in tuning monolayer structures and using them as efficient drug delivery systems. Therapeutic self-assembled monolayers (TSAMs) were prepared by chemically attaching HS-AB to gold surfaces. Their properties were studied by voltammetry and atomic force microscopy (AFM). A gold electrode with immobilized thioglycolic acid (HS-GA) was used for comparison. The surface concentration of HS-AB on the gold surface was 0.572 nmol/cm^2^, determined from the area of the voltammetric reduction peaks (desorption process). The area per one molecule estimated from the voltammetry experiments was 0.291 nmol/cm^2^. The capacity of thus prepared electrode was also tested. The calculated capacity for the HS-AB modified electrode is 2.90 μF/cm^2^. The obtained value indicates that the monolayer on the gold electrode is quite well ordered and well-packed. AFM images show the formation of gold nanoparticles as a result of immersing the HS-AB modified gold electrode in an aqueous solution containing 1 mM HAuCl_4_·3H_2_O. These structures arise as a result of the interaction between the HS-AB compound adsorbed on the electrode and the AuCl_4_^−^ ions. The voltammetric experiments also confirm the formation of gold structures with specific catalytic properties in the process of oxygen reduction.

## 1. Introduction

**Figure 1:**
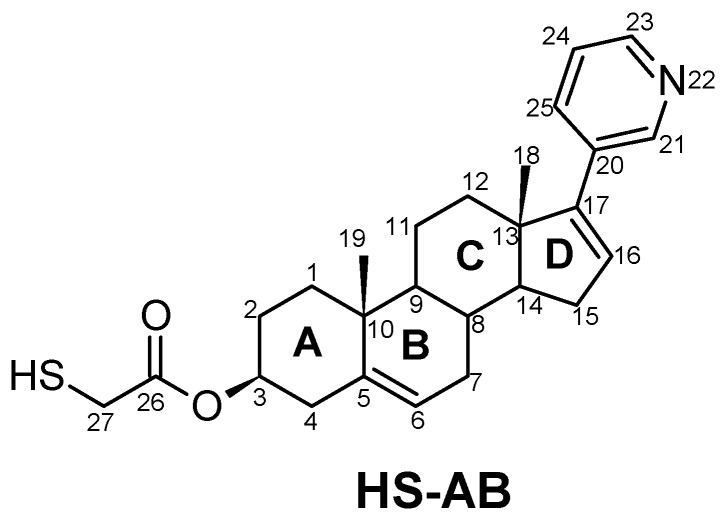
The structure of thiolated abiraterone. Four rings of the androsta-5,16-dien-3β-ol moiety are marked as A, B, C, and D.

Abiraterone is a steroidal compound with antiandrogen activity. It inhibits the enzymatic activity of steroid 17alpha-monooxygenase (the 17alpha-hydrolase/C17,20 lyase complex; CYP17A1). This enzyme is expressed in testicular, adrenal and prostatic tumor tissues and required for the androgen biosynthesis. Its administration may suppress testosterone production by both the testes and the adrenals to castrate-range levels [1]. Abiraterone may also cause serious side effects. Therefore, it is important to develop a targeted transport for this drug. Our previous studies showed that abiraterone interacts weakly with gold nanoparticles [2] which turned out to be interesting carriers of the studied drug. Hence the idea of preparing a new abiraterone analogue by the targeted thiolation of the abiraterone molecule in the area that is not responsible for the biological activity of the drug. As a result, we have obtained a new analogue of thiolated abiraterone (HS-AB) not yet described in literature. The structural formula of HS-AB is presented in Figure 1.

In view of recent advances in applying monolayer structures as efficient drug and biomolecule carriers [3,4,5], we have also focused on designing a gold surface monolayer. Self-assembled monolayers (SAMs) on gold and titanium are used in targeted drug delivery [6]. The study suggests that self-assembled monolayers could potentially be used as an alternative system for delivering drugs from coronary stents and other metal implants. The article describes the use of SAMs as a novel technique of attaching and releasing therapeutic molecules directly from the metal surfaces. Ko at al. [7] propose a cell chip with thiolated chitosan self-assembled on a gold electrode which could be used for various purposes, including discriminating normal cells from cancer cells, evaluating the efficiency of newly developed drugs and assessing the cytotoxicity of various chemicals.

The monolayers are obtained by way of a spontaneous adsorption of the molecules from a solution or gas phase on solid surfaces or by the formation of regular structures on liquid surfaces. Compounds containing thiol groups, for example, have a high affinity for metal surfaces. In recent years, a great deal of work has been devoted to the alkanethiol monolayers adsorbed on gold. Alkanethiols, sulphides and disulfides have the ability to form stable, tightly packed monolayers with a specific composition, structure and thickness. Well-defined alkanethiol monolayers on solid substrates allow modeling and testing of the processes occurring at the interface, as well as of the interactions that occur between different molecules in the monolayer [8,9,10]. Monolayers composed of specially designed organothiol molecules can also exhibit membrane, ion exchange or catalytic properties. Research on the properties of thus covered surfaces is aimed at using appropriately designed molecular layers as the components of biosensors and biocatalytic surfaces as well as in drug release systems, etc.

Among the methods used to assess the structure and properties of the electrodes modified with alkanethiol monolayers, electrochemical methods were used first. They allow to establish the presence or absence of potential defects in the monolayers. The molecules of the organothiol compounds adsorbed on the gold substrate cause a significant reduction in the capacitive currents of the electrodes, in comparison to the currents obtained on the unmodified electrodes. Non-electroactive monolayers adsorbed on the electrode form a barrier between the ions/compounds present in the solution and the surface of the electrode. When the adsorbed monolayers do not contain any defects, they form a good system for studying the kinetics of electron tunneling. Blocking properties of the monolayers adsorbed on the electrode can be tested by observing the oxidation and reduction processes of the electroactive compounds in a solution, the so-called redox probes e.g., Ru(NH_3_)_6_^2+/3+^, Fe(CN)_6_^3−/4−^, as well as by observing the anodic oxidation of the gold surface [11,12]. Such systems do not tend to adsorb to the surface of the electrodes and the kinetics of the electron transfer is relatively fast. Well-packed monolayers block the course of these processes. Monolayers with the compounds that can reduce precious metal ions such as gold can be used to create molecular interactions [13].

We have prepared therapeutic self-assembled monolayers (TSAMs) by chemically attaching the HS-AB to gold surfaces. The properties of the HS-AB modified electrodes were characterized. An electrode with immobilized HS-GA (thioglycolic acid) was used for comparison because HS-GA was a linker in the HS-AB synthesis. The self-assembly properties of the HS-GA and HS-AB compounds as well as their electrochemical properties in the supporting electrolyte itself, the electrooxidation of the fast probe, [Fe(CN)_6_]^4−^ and the desorption process were also studied. The formation of the HS-AB monolayer on the gold surface and of the gold nanoparticles on the HS-AB monolayer after immersing the HS-AB modified gold electrode in an aqueous solution containing 1 mM HAuCl_4_·3H_2_O for 24 and 72 h was characterized using atomic force microscopy (AFM). This technique confirmed the attachment of the drug to the gold surfaces of the electrode as well as the formation of gold structures, including gold nanoparticles, which have specific catalytic properties in the process of oxygen reduction. The oxygen reduction on the gold electrodes modified with HS-AB or HS-AB covered with gold nanoparticles was compared.

## 2. Materials and Methods

### 2.1. Materials

All reagents used in the study were commercially available and of the highest purity. They were obtained from POCh (Gliwice, Poland) and Sigma-Aldrich (Saint Louis, MO, USA) and used without prior purification. The water used to make the solutions and wash the electrodes was distilled and cleaned in a “Milli-Q” filter apparatus (Millipore Corporation, Bedford, MA, USA). Its final resistance was 18.2 MΩ/cm. Deuterated chloroform was purchased from ARMAR AG (Döttingen, Switzerland).

The pH of the solutions used in the experiments was determined using a commercially available Mettler Toledo (Greifensee, Switzerland) pH meter. To prepare the phosphate buffer, monobasic and dibasic sodium phosphate (V) was used. The solutions were deoxygenated or oxygeneted with argon or oxygen for 20 min before each experiment, while during the measurements gas was passed over the solution (argon and oxygen with 99.5% purity from Air Products (Kielce, Poland).

The monolayers of the HS-AB and HS-GA compounds on the gold electrodes were prepared in the self-assembly process. First, the monolayers of the HS-AB and HS-GA compounds were prepared on a gold surface by immersing the purified gold electrodes in ethanolic solutions containing 1 mM HS-AB or HS-GA. After their removal, the electrodes were rinsed thoroughly with ethanol and water to wash off the physically adsorbed adsorbate molecules and left to dry in the air.

### 2.2. Electrochemical Measurements

Electrochemical measurements were performed using an Eco Chemie Autolab POSTAT20 potentiostat (Utrecht, The Netherlands) which cooperated with a PC computer. All experiments were carried out in 22 ± 2 °C. A three-electrode system was used for the experiments, in which the reference electrode was a silver-silver chloride one (Ag/AgCl KClsat) and the counter-electrode was a platinum foil. As working electrodes, electrodes (Arrande, Werther, Germany) with the surface area of 0.61 cm^2^ were used. Substrates with vapor-deposited gold were borosilicate glass plates measuring 1.1 × 1.1 cm^2^, on which gold was deposited, 200–300 nm thick. Between the gold layer and the glass there was an adhesive layer of chromium, 2–5 nm thick. These substrates were used in the electrochemical studies as the working electrodes.

### 2.3. Atomic Force Microscopy (AFM)

The AFM measurements were carried out with a 5500 AFM instrument (Keysight Technology, Santa Rosa, CA, USA). The images were collected in a non-contact mode using SSS-NCLR silicon probes (Nanosensors nominal spring constant 21–98 N/m, resonance frequency in air in the range of 146–236 kHz). All experiments were performed in 23 ± 1 °C. For the AFM studies, the monolayer of HS-AB was immobilized on a gold single crystal Au(111) substrate purchased from MaTeck (Juelich, Germany). Before deposition the substrate was carefully flame annealed and then cleaned in the piranha solution (concentrated H_2_SO_4_/30% H_2_O_2_ 3:1, *v*/*v*) for at least 2 h. Next, it was rinsed with Milli-Q ultrapure water. The quality of the substrate was checked by AFM imaging. The HS-AB self-assembled monolayer was prepared according to the procedure set out in Section 2.1.

### 2.4. Nuclear Magnetic Resonance (NMR) Spectroscopy

All NMR measurements were performed on a Bruker Avance III HD spectrometer at the 500 MHz (Bruker Corporation, Billerica, MA, USA) transmitter frequency for 1H. The spectra were measured in the temperature of 295 K in the CDCl_3_ solution relatively to the TMS signal as a 1H and 13C chemical shift standard. The NMR studies used the results from one and two-dimensional NMR spectroscopy: 1H, 13C, HSQC, HMBC and COSY. All signals from the 1H and 13C NMR spectra were ascribed to the proper position of each atom.

### 2.5. MS Spectrometry

The ESI-MS spectra were recorded on a PE Biosystems Mariner mass spectrometer.

### 2.6. Chromatography

The progress of the reaction was monitored by thin layer chromatography (TLC) with a Merck DC-Alufolien Kieselgel 60 F_254_. The chemicals and solvents were purchased from Fluke company. Column chromatography was performed on Merck silica gel 60 (230–400 mesh).

### 2.7. Chemistry

***Abiraterone Tritylmercaptoacetate* (2)**. Abiraterone (1 g, 2.86 mM) and Tr-S-CH_2_-COOH (1.91 g, 5.72 mM) were dissolved in 30 mL dichloromethane. Then, DMAP (105 mg, 0.858 mM) was added to the solution. EDCI (1.64 g, 8.58 mM) was added dropwise to the clear solution and the reaction was stirred for 2 h at RT (TLC monitoring: hexane-ethyl acetate 1:1). The mixture was treated with 20 mL of water, next the organic solution was washed with 0.1 M NaOH and brine. The organic phase was dried over anhydrous magnesium sulphate, filtered and concentrated to the crude solid. The crude product was purified by column chromatography (hexane/ethyl acetate 5:1 1:1) to give 98% (1.88 g) of 2 as a white solid. mp 184 °C. 1HNMR (500 MHz, CDCl3): 1.09 (3H, s, CH3-18), 1.10 (3H, s, CH3-19), 1.11 (1H, m, CH-9), 1.16 (1H, m, CH2-1), 1.53 (1H, m, CH2-12), 1.58 (1H, m, CH2-2), 1.63 (1H, m, CH-14), 1.69 (2H, m, CH2-11), 1.72 (1H, m, CH2-7), 1.80 (1H, m, CH-8), 1.85 (1H, m, CH2-2), 1.89 (1H, m, CH2-1), 2.08 (1H, m, CH2-12), 2.10 (1H, m, CH2-7), 2.10 (1H, m, CH2-15), 2.31 (1H, m, CH2-15), 2.32 (2H, m, CH2-4), 2.98 (2H, s, CH2-27), 4.57 (1H, m, CH-3), 5.44 (1H, d, (*J* = 4.4 Hz), CH-6), 6.04 (1H, m, CH-16), 7.26 (1H, m, CH-24), 7.27 (3H, m, CH-32), 7.34 (6H, dd, (*J*1 = 7.9; *J*2 = 7.3 Hz), CH-31), 7.47 (6H, d, (*J* = 7.5 Hz), CH-24), 7.69 (1H, dt, (*J*1 = 7.9; *J*2 = 1.8 Hz), CH-25), 8.51 (1H, dd, (*J*1 = 4.8; *J*2 = 1.4 Hz), CH-23), 8.67 (1H, d, (*J* = 1,6 Hz), CH-21); δ, 13C NMR (125 MHz, CDCl3): 16.54 (CH3-18), 19.21 (CH3-19), 20.76 (CH2-11), 27.48 (CH2-2), 30.34 (CH-8), 31.46 (CH2-7), 31.75 (CH2-15), 35.03 (CH2-27), 35.14 (CH2-12), 36.70 (C-10), 36.78 (CH2-1), 37.84 (CH2-4), 47.27 (C-13), 50.15 (CH-9), 57.40 (CH-14), 67.05 (C-28), 74.92 (CH-3), 122.39 (CH-6), 122.99 (CH-24), 126.83 (CH-32), 127.99 (CH-31), 129.19 (CH-16), 129.53 (CH-30), 132.90 (C-20), 133.64 (CH-25), 139.80 (C-5), 144.07 (C-29), 147.85 (CH-23), 147.89 (CH-21), 151.61 (C-17), 169.00 (C-26).; HR-MS (ES+) calcd. for C_45_H_48_NO_2_S (M + H)^+^: 666.3406. Found: 666.3392.

***Abiraterone 2-mercaptoacetate* (3)**. Tr-S-Abirateron (775 mg, 1.2 mM) was dissolved in 10 mL dichloromethane under argon atmosphere, and cooled to 0 °C (an ice bath). The colourless solution was treated with 2 mL of trifluoroacetic acid, and next triethylsilane (1 mL) was immediately added to the reaction mixture. The reaction was monitored by TLC (hexane:ethyl acetate:methanol 5:3:1) and completed after 20 min. Then the reaction was quenched by triethylamine (1 mL) and the solution was washed with water (20 mL) and brine (20 mL). The organic phase was evaporated to oil in room temperature. The crude oil was purified by column chromatography (hexane:ethyl acetate:methanol 5:3:1) to afford 81% (412 mg) of 3 as a white solid. m.p. 151 °C. 1HNMR (500 MHz, CDCl3): 1.04 (3H, s, CH3-18), 1.07 (1H, m, CH-9), 1.08 (3H, s, CH3-19), 1.16 (1H, m, CH2-1), 1.48 (1H, m, CH2-12), 1.58 (1H, m, CH-14), 1.61 (1H, m, CH2-2), 1.62 (2H, m, CH2-11), 1.67 (1H, m, CH2-7), 1.75 (1H, m, CH-8), 1.87 (1H, m, CH2-1), 1.89 (1H, m, CH2-2), 1.99 (1H, t, (*J* = 8.2 Hz), SH), 2.03 (1H, m, CH2-12), 2.06 (1H, m, CH2-7), 2.06 (1H, m, CH2-15), 2.27 (1H, m, CH2-15), 2.37 (2H, m, CH2-4), 3.23 (2H, d, (*J* = 8.2 Hz), CH2-27), 4.65 (1H, m, CH-3), 5.43 (1H, d, (*J* = 5.1 Hz), CH-6), 6.01 (1H, m, CH-16), 7.25 (1H, dd, (*J*1 = 8.0; *J*2 = 4.5 Hz), CH-24), 7.69 (1H, dt, (*J*1 = 8.0; *J*2 = 1.9 Hz), CH-25), 8.46 (1H, dd, (*J*1 = 4.8; *J*2 = 1.5 Hz), CH-23), 8.62 (1H, d, (*J* = 2.0 Hz), CH-21); δ, 13C NMR (125 MHz, CDCl3): 16.53 (CH3-18), 19.21 (CH3-19), 20.76 (CH2-11), 26.82 (CH2-27), 27.51 (CH2-2), 30.32 (CH-8), 31.45 (CH2-7), 31.77 (CH2-15), 35.11 (CH2-12), 36.71 (C-10), 36.78 (CH2-1), 37.87 (CH2-4), 47.27 (C-13), 50.14 (CH-9), 57.39 (CH-14), 75.19 (CH-3), 122.52 (CH-6), 123.22 (CH-24), 129.64 (CH-16), 133.19 (CH-25), 134.22 (C-20), 139.68 (C-5), 147.15 (CH-23), 147.19 (CH-21), 151.34 (C-17), 170.27 (C-26).; HR-MS (ES+) calcd. for C_26_H_33_NO_2_S (M + H)^+^: 424.2310. Found: 424.2307.

***2-(tritylthio)acetic acid* (4)**. Mercaptoacetic acid (0.35 mL, 5 mM) and trityl chloride (1.395 g, 5 mM) were dissolved in 3 mL of acetic acid and 3 mL of dichloromethane. Next, BF3*OEt (1 mL) was added and the reaction mixture was stirred for 1 h at RT. The solvents were evaporated and water (5 mL) was added to the residue. The white solid was filtered off and the title compound **4** (1.65 g) was obtained with a 98% yield. m.p.: 165 °C. NMR consistent with literature data [14]. HR-MS (ES−) calcd. for C_21_H_17_O_2_S (M–H)^−^: 333.0949. Found: 333.0941.

## 3. Results and Discussion

### 3.1. Chemistry

**Scheme 1:**
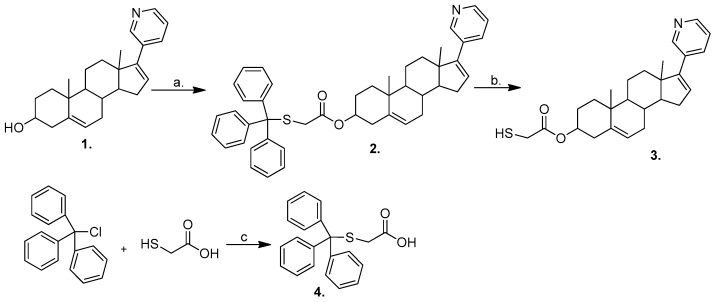
Reagents and conditions: (**a**)—Tr-SCH_2_COOH **4**, EDCI, DMAP, rt (**b**)—TFA, Et_3_SiH, NEt_3_ (**c**)—AcOH, BF_3_·Et_2_O.

The synthesis of abiraterone 2-mercaptoacetate **3** is presented in Scheme 1. We started our synthetic experiments with an attempt at a direct esterification of 2-mercaptoacetic acid with commercially available abirateron **1**. Common reagents from the peptide chemistry: EDCI and DCC were tested, with no positive results. It was assumed that the main cause of failure was the presence of a free thiol group in the organic acid substrate. Therefore, the thiol group in 2-mercaptoacetic acid was protected with a trityl group in reaction with trityl chloride. The esterification of 2-(tritylthio)acetic acid **4** with abiraterone, using EDCI as the coupling reagent, gave ester 2 with a 98% high yield following purification. The final deprotection by the trifluoroacetic acid (TFA)/triethylsilane (Et_3_SiH) system in room temperature and under nitrogen atmosphere gave abiraterone 2-mercaptoacetate **3** with an 81% yield. The structures of new compounds **2** and **3** were confirmed by extended 1D and 2D NMR experiments, as well as by HRMS.

### 3.2. Electrochemical and AFM Characteristics of the Gold Electrodes Modified with HS-AB or HS-GA

The electrochemical measurement of the capacity is a method used relatively frequently to verify the quality of the monolayers. At the interface between the metal and the electrolyte solution there is a so-called double electrical layer which can be simply described as a capacitor with oppositely charged covers, one cover being a metal surface and the other a fixed layer of ions from the solution adhering to the metal (Helmholtz model). When using electrochemical methods, it is possible to determine the capacity of this conventional capacitor, i.e., a double electric layer. Capacity is one of the basic parameters characterizing monolayers in terms of their quality. On the basis of the capacitive measurements we can determine to what extent the monolayer is penetrated by water or the ions. The capacity for the unmodified gold electrode is usually about 10 to 80 μF/cm^2^ [15,16,17], while for the electrodes coated with a well-organized monolayer of long-chain alkanethiols, values in the range of 1–2 μF/cm^2^ [18,19] are obtained. Indirect capacitance values indicate the presence of defects in the monolayer. A defect should be understood as an area on the surface of a modified electrode, where the monolayer can be penetrated by electrolyte ions or solvent molecules. The higher the value of the measured capacity, the more defects there are in the monolayers. Alkanethiol molecules adsorbed on the gold surface cause a significant decrease in the capacitive currents. This effect is related to the low value of the dielectric constant of the layer separating the electrode metal from the solution. As a result, the measured capacity is an order of magnitude lower than that obtained for the unmodified gold electrodes. Small capacitance values indicate low permeability of the monolayer for the basic electrolyte ions and water, which suggests a good arrangement of the systems [11,12,18,20]. If the capacity value falls below 1 μF/cm^2^, then it may suggest a possibility of the multilayers’ formation in the case of alkanethiol modified electrodes.

**Figure 2:**
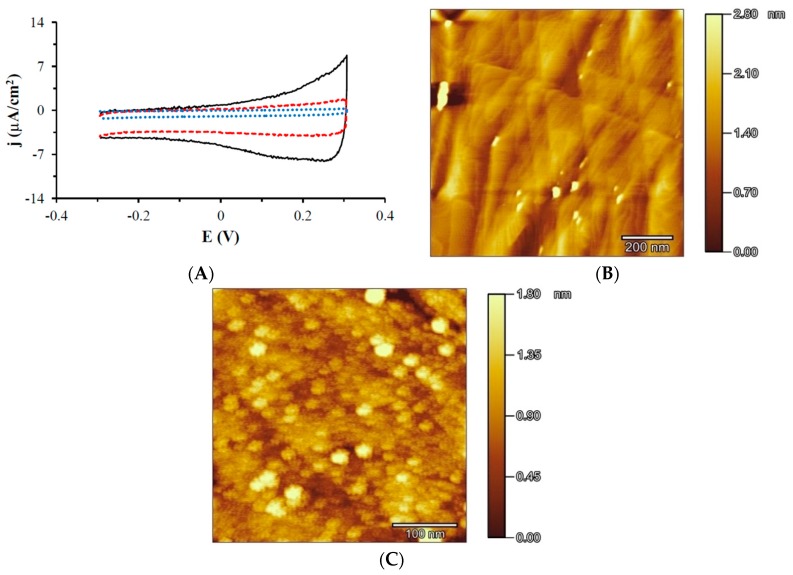
(**A**) Cyclic voltammetric curves recorded using gold electrodes unmodified (black continuous line curve) and modified with HS-GA (red curve short-dashed line) or HS-AB (blue curve dotted line) in 0.2 M of the phosphate buffer (pH 7.0), the scan rate of the potential 0.1 V/s. AFM images of gold (**B**) unmodified, (**C**) modified with the HS-AB compound.

In our research, we used HS-AB and HS-GA compounds to modify gold electrodes in the self-assembly process. First, the monolayers of the HS-AB and HS-GA compounds were prepared on a gold surface by immersing the purified gold electrodes in ethanolic solutions containing HS-AB or HS-GA. After their removal, the electrodes were rinsed thoroughly with ethanol and water to wash off the physically adsorbed adsorbate molecules and left to dry in the air. The prepared electrodes were then examined by cyclic voltammetry in the deoxygenated 0.2 M phosphate buffer (pH 7.0). Voltammetric curves were recorded in the potential range from −0.3 V to 0.3 V at the scan rate of the potential: 0.1 V/s for the gold electrodes unmodified and modified with HS-AB or HS-GA (Figure 2). No peaks were observed on the voltammetric curves, only the capacitive currents associated with the charging and discharging capacitors, i.e., gold electrodes unmodified or modified with thiol compounds. The value of the electrodes’ capacitance was calculated on the basis of the capacitive currents measured from the voltammetric curves at the potential of 0 V [12,17]. The capacities of individual systems are respectively: for the unmodified gold electrode 32.31 ± 0.67 μF/cm^2^, 18.51 ± 0.54 μF/cm^2^ for the gold electrode modified with HS-GA and 2.90 ± 0.15 μF/cm^2^ for the gold electrode modified with HS-AB. Small capacitance values for HS-AB indicate low monolayer permeability for the ions of the supporting electrolyte and water. This suggests a fairly good interaction between the AB parts and a fairly good arrangement of the systems at the molecular level, but not as good as for the long-chain alkanethiols. In the HS-AB monolayer a defect is most likely present. This is related to the construction of the HS-AB molecule in which we can distinguish a short HSCH_2_COO^−^ link anchoring the AB compound to the Au surface. A low capacity value for HS-AB suggests that AB molecules do not lie on the surface, but are placed in parallel, which causes the monolayer to be compact. The packing monolayer of the HS-AB is better than that of HS-GA, because the capacity value for the HS-GA modified electrode is much larger. HS-GA, due to its structure (one CH_2_ group and one COOH group in the molecule) forms poorly ordered monolayers. In addition, the presence of a carboxyl group is not conducive to the formation of a well-arranged monolayer. Chidsey and Loiacono [20] have shown that the presence of end groups such as -CH_2_OH, -CN or -COOH in long-chain alkanethiols increases the capacity of the electrode from the capacities close to 1 µF/cm^2^ up to about 4 µF/cm^2^.

AFM measurements were also carried out for unmodified gold and HS-AB modified gold. Figure 2B shows an image recorded using atomic force microscopy for a bare gold substrate in the absence of HS-AB molecules. The average surface roughness (Sq) determined from the 0.5 × 0.5 µm^2^ scan area was 0.015 nm, which confirms good quality of the substrate. Figure 2C presents an AFM image of the same substrate recorded after a 24 h immersion in the HS-AB solution. Under such conditions the HS-AB molecules form an adlayer on the Au(111) surface. As can be seen, numerous micellar aggregate boundaries are visible as bright spots. The HS-AB forms a well packed monolayer, because AB is a rather flat molecule. Its structure is similar to that of cholesterol [21]. Cholesterol is intensely studied. It forms well-packed monolayers on various substrates, which is confirmed, for example, by AFM, just as it happened in our study [22,23,24].

In our research, the capacity of the HS-GA electrode is much larger than that of the HS-AB modified electrode, but smaller in comparison to the unmodified gold electrode. Short-chain compounds of alkanethiols with the COOH groups [16,17,25,26,27,28,29], which are easy to modify, are very often used as systems in order to attach other compounds, proteins and enzymes to the gold electrode. In our research we used them to attach abiraterone to the gold surface.

### 3.3. Electrochemical Characteristics of Gold Electrodes Modified with HS-AB or HS-GA in the Presence of the Redox Probe Fe(CN)_6_^4−^

**Figure 3:**
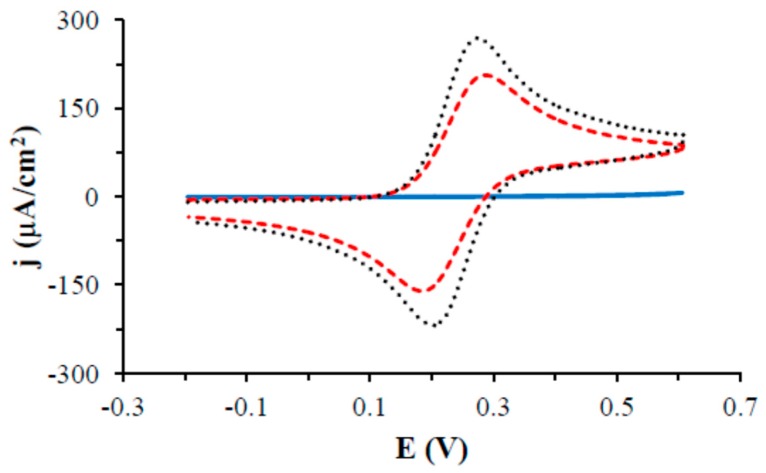
Cyclic voltammetric curves recorded using gold electrodes unmodified (black curve dotted line) and modified with HS-GA (red curve short-dashed line) or HS-AB (blue curve continuous line) in deoxygenated 0.5 M KCl containing 1 mM Fe(CN)_6_^4−^, the scan rate of the potential 0.1 V/s.

The next step in the process of checking the quality of the monolayers on gold was to examine the HS-AB or HS-GA modified electrodes in the presence of a fast redox probe Fe(CN)_6_^4−^ in a solution. The tests were carried out in deoxygenated 0.5 M KCl containing 1 mM Fe(CN)_6_^4−^. Figure 3 shows the voltammetric curves registered using the unmodified and HS-AB or HS-GA modified gold electrodes in the potential range from −0.2 V to 0.6 V at the scan rate of the potential of 0.1 V/s. The curve obtained for the HS-AB modified electrode showed only an increase in the oxidation current with the increasing overpotential, suggesting a kinetic control of the electrode process. The exponential shape of the voltammetric curve indicates good blocking properties of the HS-AB monolayer. Different properties are observed for the monolayer formed by the HS-GA compound. On the voltammetric curve, as for the unmodified gold electrode, oxidation and reduction peaks of the redox probe are observed. Peak potentials and currents from the curves recorded using the unmodified and HS-GA modified gold electrodes were measured and the results are shown in Table 1. The peak potential of the oxidation peak is much more positive and the reduction potential of the redox probe is much more negative for the HS-GA modified gold electrode as compared to the unmodified Au electrode. The calculated difference between the peak potential of the oxidation and reduction peaks of the redox probe is also greater for the HS-GA-modified gold electrode as compared to the unmodified electrode. Moreover, the value of the oxidation peak current and reduction of the redox probe for the HS-GA electrode is smaller in comparison to that of the unmodified electrode. This indicates a slight blockage of the redox probe transport to the electrode surface through the adsorbed HS-GA monolayer. The presence of a carboxylic group exposed to the solution in the HS-GA monolayer, which under these conditions is negatively charged (p*K*a (COOH) = 3.55) [30], repels the electrostatically negatively charged redox probe. The result is a slight decrease in the oxidation and reduction currents, as well as an increase in the difference of the potentials between the oxidation and reduction peaks of the redox probe in comparison to the gold electrode. This is consistent with literature [27,29]. The presence of negatively charged COO^−^ groups does not increase the order of the monolayer, because there is an electrostatic repulsion of the negatively charged carboxylic groups in the HS-GA multiplication on the surface of the gold electrode. The influence of carboxyl groups in alkanethiols on the organized monolayers was studied by Chidsey and Loiacono [20]. Based on their spectroscopic measurements, the authors found that the monolayers of alkanethiols containing terminal carboxylic groups are quasi-liquid. These results indicate that a monolayer of alkanethiols with terminal carboxylic groups exhibits permeability to ions and water molecules, which is consistent with its quasi-liquid nature. Therefore, the redox probe reaches the gold surface almost freely. The presence of only one CH_2_ group in HS-GA does not provide a sufficiently strong interaction between the CH_2_ groups of the compound to effectively impede the access of the redox probe to the gold electrode surface.

HS-AB has different properties in comparison to HS-GA. The layer is compact and fairly well-organized, which is manifested by the lack of the oxidation and reduction signals of the probe in the tested range of the potentials. This suggests strong interactions between the AB AB groups. Similar conclusions about quite well-organized monolayers were drawn based on studying the capacities of modified electrodes (see previous paragraph).

### 3.4. Electrochemical Characteristics of Gold Electrodes Modified with HS-AB or HS-GA in the NaOH Solution

**Figure 4:**
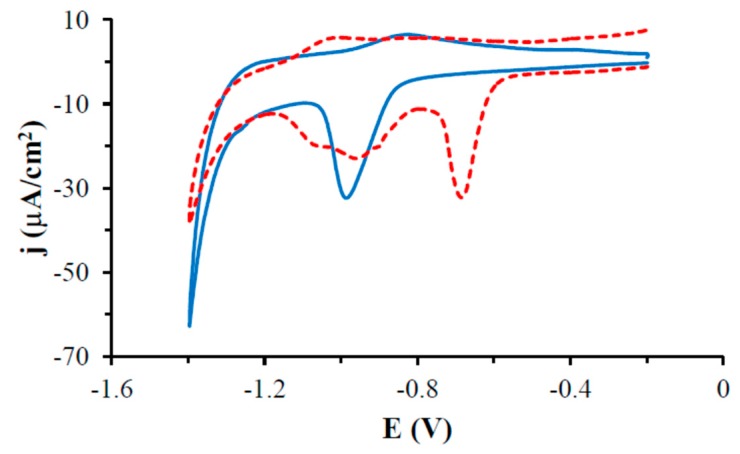
Cyclic voltammetric curves recorded in deoxygenated 0.1 M NaOH using gold electrodes modified with HS-GA (red curve short-dashed line) or HS-AB (blue curve continuous line), the scan rate of the potential 0.05 V/s.

The properties of the monolayer can be investigated by the electrochemical desorption process [31,32,33]. The HS-AB or HS-GA modified gold electrodes were examined electrochemically in deoxygenated 0.1 M NaOH and voltammetric curves were recorded in the potential range from −0.2 V to −1.4 V (Figure 4). On the voltammetric curves the reduction peak as well as the oxidation peaks were observed. For the HS-AB modified electrode the reduction peak was observed at the potential of −0.984 V and the oxidation peak at the −0.830 V potential. The reduction peak corresponds to the desorption of HS-AB thiols from the gold surface. This process is not irreversible, however, because the application of a more positive potential than the desorption potential causes again the adsorption of the thiol molecules on the metal surface, we can thus observe a peak at the −0.830 V potential corresponding to the adsorption of thiol on the gold electrode surface. The adsorption peak, however, is much smaller than the desorption peak, which is due to the fact that some of the previously desorbed thiol molecules diffuse into the solution, so there is no possibility of them being re-adsorbed to the electrode surface. For the HS-GA-modified gold electrode the reduction peaks were interrupted at the potential of −0.660 V and ca. −0.940 V, there was also one oxidation peak at the potential of −1.050 V. The reduction peaks correspond to the desorption of the HS-GA thiol molecules from the gold surface. In the case of the HS-GA compound, desorption occurs in two stages, as evidenced by more than one reduction peak. This is related to the occurrence of two kinds of an interaction: between the CH_2_ CH_2_ groups and between the carboxyl groups. Schilardi et al. report that electrodesorption occurs in one peak and at a more negative potential whereas the van der Waals interaction is very strong as it occurs in long-chain alkanethiols [34]. In our research the HS-AB electroreduction occurs at more negative potentials and in one peak. The HS-GA desorption peak is at a lower potential than the HS-AB reduction peak, which corresponds to our observation that the HS-AB molecules interact more strongly with one another in comparison to HS-GA. Imabayashi et al. also state that due to the repulsive interaction between the negatively charged carboxylate groups in SAM, the reductive desorption peak of COOH(CH_2_)_n_SH is more positive than that of CH_3_(CH_2_)_n_SH (n—numbers of CH_2_ groups) [32]. The charge under the desorption peak of the HS-AB and HS-GA monolayer was measured and the results are shown in Table 2. The surface concentration of the monolayer was also calculated, as well as was the area per one molecule [12,13].

The charge calculated under the HS-GA desorption peaks is about 37% larger in comparison to HS-AB, and the area per one molecule is 27% lower. The HS-GA surface concentration is 37% higher than that of HS-AB, but this does not guarantee a well-organized monolayer. This is related to the construction of the HS-GA molecule and the interactions in the monolayer, as described in previous chapters. For well-ordered thiol monolayers, the surface concentration should be 0.78 ± 0.06 nmol/cm^2^ which corresponds to the average area per one molecule equal about 0.20 nm^2^. However, it should be remembered that thus determined surface concentration is burdened with an error resulting from loading a double layer during the desorption process. This is due to the fact that during the desorption process the capacity varies from 1–2 μF/cm^2^ to about 20 μF/cm^2^. Therefore, the surface concentration evaluated in the above manner is usually overestimated. No literature data on the abiraterone monolayer or the surface area per one molecule has been found. Because the abiraterone molecule has a similar structure to that of cholesterol, the result obtained was compared with those published for the cholesterol monolayer. The most common technique for creating cholesterol monolayers is the Langmuir-Blodgett (L-B) technique which allows to determine the area per one molecule. The area determined per one molecule of cholesterol using the L-B technique fluctuates from 0.30 to 0.60 nm^2^ [22,23,24,35,36]. Similar information was obtained from theoretical studies. In our research, the surface area per HS-AB molecule is about 0.30 nm^2^, which is close to the results described for cholesterol.

### 3.5. Electrochemical Characteristics of Gold Electrodes Modified with HS-AB or HS-GA in the Solution Containing Oxygen

**Figure 5:**
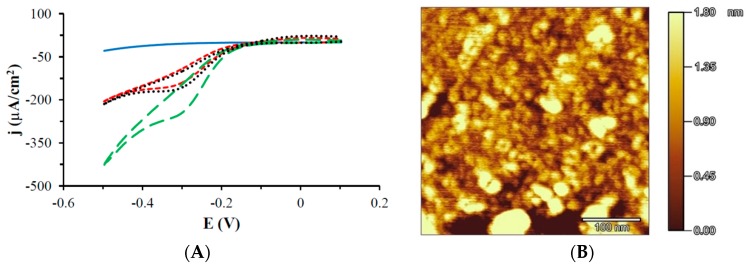

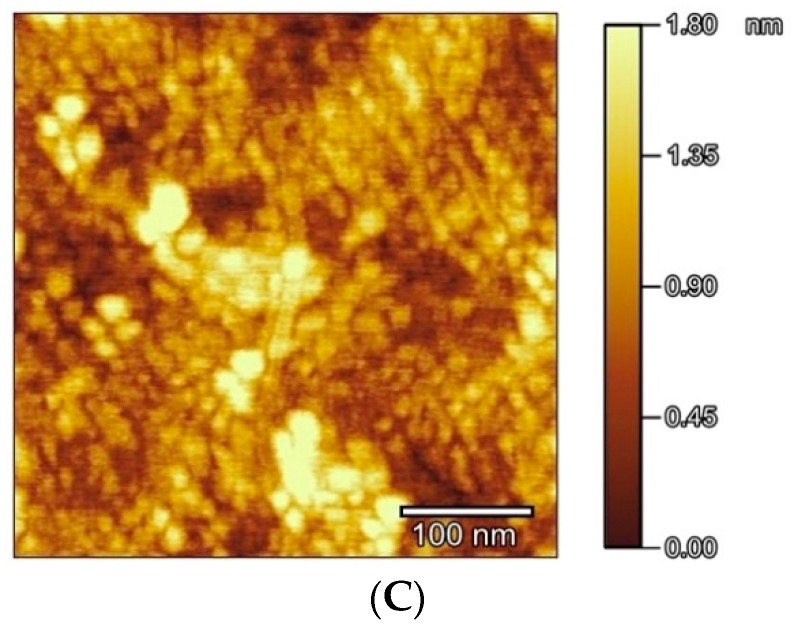
(**A**) Cyclic voltammetric curves recorded in oxygenated 0.5 M KCl using an unmodified (black curve dotted line) or gold electrode modified with HS-GA (red curve short-dashed line), HS-AB (blue curve continuous line) and HS-AB after immersing in the 1mM AuCl_4_^−^ aqueous solution for 24 h (green curve long-dashed line), the scan rate of the potential 5 mV/s. AFM images of gold modified with the HS-AB compound after immersing for (**B**) 24 h; (**C**) 72 h in the solution containing 1 mM AuCl_4_^−^.

The oxygen reduction process was tested using unmodified as well as HS-GA or HS-AB modified gold electrodes. It is well known that gold catalyzes the oxygen reduction process [37,38,39]. First, the oxygen reduction process was tested on the unmodified gold electrode (Figure 5). On the voltammetric curve, a peak of the oxygen reduction at the potential of −0.337 V and a density oxygen reduction current −164.2 μA/cm^2^ (Table 3) were observed in the tested potential range. After modifying the gold electrode with the HS-AB compound, no oxygen reduction peak was observed in the tested potential range. Research suggests that the layer is impermeable to molecular oxygen, so it is fairly well-organized. The HS-GA monolayer is oxygen-permeable, therefore there is a visible oxygen reduction peak at the −0.353 V potential similar to that of the unmodified gold electrode. Only a slight decrease in the density reduction current of oxygen −152.1 μA/cm^2^ is observed on the gold electrode modified with HS-GA as compared to the unmodified gold electrode, suggesting a slight decrease in oxygen diffusion to the electrode surface through the HS-GA monolayer. After immersing the HS-AB modified gold electrode in AuCl_4_^−^ gold ions, the oxygen reduction peak was observed at the −0.308 V potential. The peak is at a less negative potential than that of the unmodified electrode and the density current of the oxygen reduction −248.8 μA/cm^2^ is higher than that for the electrode unmodified (by about 51%) and modified with HS-GA (by about 64%). This suggests a catalytic process of oxygen reduction on the gold structures emerging on the AB monolayer (Au/HS-AB/ AuNp). The study also confirms our earlier research, where we had described the activity of a pyridine group in the formation of gold nanoparticles [2]. That study also suggested that the pyridine group is an active group of AB, which is consistent with the cytotoxic studies.

The hypothesis of gold structures being formed in the presence of the HS-AB compound was verified by the AFM method. Figure 5B,C show images recorded after immersing gold modified with HS-AB in an aqueous solution containing 1 mM HAuCl_4_·3H_2_O for 24 h and 72 h, respectively. The obtained images clearly show more gold nanoparticles. There are more of them after a 72 h exposure of the HS-AB modified electrodes to AuCl_4_^−^ ions. The gold nanoparticles are arranged in a characteristic pattern of parallel rows and their diameter is around 20 nm. All gold nanoparticle structures arise as a result of the interaction of the HS-AB compound adsorbed on the electrode with the AuCl_4_^−^ ions. As the pyridine groups are exposed to the solution, one can conclude that they are responsible for the reduction of gold ions. Literature describes methods of obtaining gold nanoparticles from the compounds containing nitrogen atoms in their molecules in the presence of the reducing agent NaBH_4_ [40,41,42,43]. Đurovic and et.al. report that pyridine spontaneously reduced gold ions from AuCl_4_^−^ to Au^+^, but they did not observe any formation of atomic gold or gold nanoparticles [44]. Literature also describes the possibility of a spontaneous gold nanoparticle synthesis in the living organisms, where the compounds present in their cells serve as a reducing and stabilizing factor for gold nanoparticles [45,46]. Modified gold nanoparticles are intensively used as systems for the preparation of sensors [47,48] but also in the construction of enzymatic biofuel cells [49,50]. Such systems using gold nanoparticles offer a possibility of determining lower concentrations of the analyte in the case of sensors and obtaining higher current and power densities for biofuel cells. Gold nanoparticles improve charge transport between a substance in a solution and the electrode surface [11,51,52].

## 4. Conclusions

Our article reports an original synthesis of a new compound: thiolated abiraterone (HS-AB). We have designed therapeutic self-assembled monolayers (TSAMs) by chemically attaching HS-AB to gold surfaces.

Small capacitance values for HS-AB indicate low permeability of the monolayer for basic electrolyte ions and water. This suggests a fairly strong interaction between the AB parts and suggests a good arrangement of the systems at the molecular level. The low capacitance value suggests that AB is a flat particle and does not lie on the surface, but is set in parallel, which results in the solidity of the monolayer. Layer ordering is better than for HS-GA, because the capacity value for the HS-GA modified electrode is much larger. This may suggest that HS-GA forms poorly ordered mono-layers. The quality of the gold monolayers was tested in the presence of the redox probe Fe(CN)_6_^4−^ in a solution. The curves obtained for the HS-AB modified electrode only showed an increase in the oxidation current with the increasing overpotential, suggesting a kinetic control of the electrode process. The exponential shape of the voltammetric curves shows good blocking properties of the HS-AB monolayer. The layer is compact and well organized as evidenced by the absence of the oxidation signals and reduction of the probe in the tested potential range. This suggests a strong interaction between AB and AB species. The AB molecules interact very well with one another. The charges were measured at the desorption peak of the HS-AB or HS-GA monolayer which is 33.39 and 45.67 μC/cm^2^, respectively. The surface concentration of the HS-AB or HS-GA monolayer was also calculated, as well as the surface area per molecule which are 0.291 and 0.213 nm^2^, respectively.

Thus, this study confirms that the self-assembled monolayers could be used as an alternative drug delivery system, e.g., from coronary stents and other metal implants. Our study is very promising from the point of view of a possible application of the HS-AB- gold nanoparticle conjugate in anticancer therapy.

## Figures and Tables

**Table 1:** Characteristics of the cyclic voltammetric curves registered in deoxygenated 0.5 M KCl containing 1 mM Fe(CN)_6_^4−^ using unmodified and HS—GA modified gold electrodes, the scan rate of the potential 0.1 V/s.

Electrode	*E*_p,a_ [V]	*I*_p,a_ [µA]	*E*_p,c_ [V]	*I*_p,c_ [µA]	*E*_p,a_ − *E*_p,c_ [V]
**Au HS-GA**	0.281 ± 0.001	125.5 ± 0.7	0.189 ± 0.003	−100.5 ± 5.0	0.092 ± 0.004
**Au**	0.273 ± 0.003	163.0 ± 1.0	0.203 ±0.001	−132.7 ± 2.2	0.070 ± 0.003

where *E*_p,a_—the anodic peak potential, *I*_p,a_—the anodic peak current, *E*_p,c_—the cathodic peak potential, *I*_p,c_—the cathodic peak current.

**Table 2:** Characteristics of the HS-AB or HS-GA modified gold electrodes after their desorption.

Monolayer	*E*_d_ [V]	*Q* [μC/cm^2^]	*Γ* [nmol/cm^2^]	*A* [nm^2^]
**HS-AB**	−0.984 ± 0.008	33.39 ± 2.31	0.572 ± 0.040	0.291 ± 0.019
**HS-GA**	−0.660 ± 0.023 −0.950	45.67 ± 2.73	0.782 ± 0.047	0.213 ± 0.013

where *E*_d_—the peak potential from the reductive desorption process, *Q*—the area under the reduction peak (desorption peak) is equal to the charge of the molecule desorption, *Γ*—the surface coverage, *A*—the molecular area.

**Table 3:** Characteristics of the oxygen reduction process on the gold electrodes unmodified and modified with HS-GA or HS-AB and the gold nanoparticles (Au/HS-AB/ AuNp).

Electrode	*E* [V]	*I* [μA/cm^2^]
**Au**	−0.337 ± 0.004	−164.2 ± 5.8
**Au/HS-GA**	−0.353 ± 0.007	−152.1 ± 3.3
**Au/HS-AB/AuNp**	−0.308 ± 0.009	−248.8 ± 10.5
